# Effects of Diesel, Heavy Metals and Plastics Pollution on Penguins in Antarctica: A Review

**DOI:** 10.3390/ani11092505

**Published:** 2021-08-26

**Authors:** Nurul Aini Puasa, Azham Zulkharnain, Gayathiri Verasoundarapandian, Chiew-Yen Wong, Khadijah Nabilah Mohd Zahri, Faradina Merican, Noor Azmi Shaharuddin, Claudio Gomez-Fuentes, Siti Aqlima Ahmad

**Affiliations:** 1 Department of Biochemistry, Faculty of Biotechnology and Biomolecular Sciences, Universiti Putra Malaysia, Serdang 43400, Selangor, Malaysia; nurulainipuasa@gmail.com (N.A.P.); gayathiri1802@gmail.com (G.V.); khadijahnabilah95@gmail.com (K.N.M.Z.); noorazmi@upm.edu.my (N.A.S.); 2 Department of Bioscience and Engineering, College of Systems Engineering and Science, Shibaura Institute of Technology, 307 Fukasaku, Minuma-ku, Saitama 337-8570, Japan; azham@shibaura-it.ac.jp; 3 School of Health Sciences, International Medical University, Kuala Lumpur 57000, Malaysia; WongChiewYen@imu.edu.my; 4 National Antarctic Research Centre, B303 Level 3, Block B, IPS Building, Universiti Malaya, Kuala Lumpur 50603, Malaysia; 5 School of Biological Sciences, Universiti Sains Malaysia, Gelugor 11800, Penang, Malaysia; faradina@usm.my; 6 Department of Chemical Engineering, Universidad de Magallanes, Avda. Bulnes, Punta Arenas 01855, Región de Magallanes y Antártica Chilena, Chile; claudio.gomez@umag.cl; 7 Center for Research and Antarctic Environmental Monitoring (CIMAA), Universidad de Magallanes, Avda. Bulnes, Punta Arenas 01855, Región de Magallanes y Antártica Chilena, Chile

**Keywords:** Antarctica, penguins, pollution, diesel, heavy metals, microplastics, toxicity

## Abstract

**Simple Summary:**

Antarctica is contaminated by anthropogenic pollution. Due to the persistent low temperatures, the toxic impacts of pollution to the environment can be extensive. The severity of the effects varies according to the animal species, chemical type and level of exposure. Penguins are at major risk as they are the most prominent group of animals in Antarctica. This review highlights the background of penguins in Antarctica, the anthropogenic pollution and cases, as well as the toxic effects of diesel, heavy metals and microplastics toward penguins. A bibliometric analysis is also included.

**Abstract:**

Antarctica is a relatively pristine continent that attracts scientists and tourists alike. However, the risk of environmental pollution in Antarctica is increasing with the increase in the number of visitors. Recently, there has been a surge in interest regarding diesel, heavy metals and microplastics pollution. Contamination from these pollutants poses risks to the environment and the health of organisms inhabiting the continent. Penguins are one of the most prominent and widely distributed animals in Antarctica and are at major risk due to pollution. Even on a small scale, the impacts of pollution toward penguin populations are extensive. This review discusses the background of penguins in Antarctica, the anthropogenic pollution and cases, as well as the impacts of diesel, heavy metals and microplastics toxicities on penguins. The trends of the literature for the emerging risks of these pollutants are also reviewed through a bibliometric approach and network mapping analysis. A sum of 27 articles are analyzed on the effects of varying pollutants on penguins in Antarctica from 2000 to 2020 using the VOSviewer bibliometric software, Microsoft Excel and Tableau Public. Research articles collected from the Scopus database are evaluated for the most applicable research themes according to the bibliometric indicators (articles, geography distribution, annual production, integrated subject areas, key source journals and keyword or term interactions). Although bibliometric studies on the present research theme are not frequent, our results are sub-optimal due to the small number of search query matches from the Scopus database. As a result, our findings offer only a fragmentary comprehension of the topics in question. Nevertheless, this review provides valuable inputs regarding prospective research avenues for researchers to pursue in the future.

## 1. Introduction

Antarctica is the most isolated continent and is located at the southernmost part of the globe. It endures extreme cold temperatures and is a popular destination for scientists, explorers and visitors. The number of visitors to the continent from around the world showed an exponential increase from 35,000 visitors in 2012–2013 to 73,991 visitors in 2019–2020 [1,2]. With this drastic increase in visitor numbers, the risks to the Antarctic environment also grows proportionally, thus increasing concerns regarding pollution in the continent [3,4].

The extreme ambient temperatures of Antarctica negatively affect the natural recovery process. Under constant low temperatures, the natural remediation processes on the continent are slower compared to those in warmer regions or the tropics. Organic chemical pollutants such as oil-based products tend to be more persistent and harder to remediate in low-temperature regions due to the slower biodegradation rate [5,6,7,8]. Hence, in the cold continent of Antarctica, oil pollutants tend to persist for a long period of time.

Anthropogenic environmental pollution in Antarctica has led to a recognition of the potential negative impacts on the organisms living there. Penguins are of particular interest to researchers due to their utility as an indicator species for pollution and climate change. Unlike many other indigenous Antarctic vertebrates, penguins are widely distributed, non-cryptic and non-secretive animals that are also easy to observe [9]. In addition, penguins are extremely sensitive to sea ice variations and are at risk due to their slow natural recovery rate and declining population. The population of chinstrap penguins (*Pygoscelis antarcticus*) has been reduced by up to 77%—from 122,550 breeding pairs in 1971 down to 52,786 in January 2020 [10]. It is suspected that pollution in Antarctica can cause disproportionately larger adverse effects toward penguins [11]. Due to the population decline and serious health effects towards penguins, this review article is timely and should be of interest to the scientific community. Moreover, Antarctica holds the largest reservoir (90%) of the world’s freshwater and exerts a huge influence on the Earth’s climate; thus, the pollution issue in Antarctica is a global concern [12,13]. In addition, this paper systematically reviews the extant literature on the topic of diesel, heavy metals and microplastics pollution in Antarctica towards penguins from 2000 to 2020. The aim of this review paper is to assess the current information and conceptualize future research trends using a bibliometric analysis, as well as to highlight research gaps that need to be addressed in order to provide valuable inputs and prospective research avenues for scientists in this field.

The use of bibliometric indicators as a framework for assessing research performance is gaining more attention among students, academicians, professionals, scientific regulators and innovation managers. Bibliometrics offer a platform that can be effortlessly scaled up from the institute level to the global scale. The implementation of a bibliometric analysis at the onset of new research can confirm that important references from the literature are taken into account when developing the research. Furthermore, bibliometric analysis can reveal knowledge gaps, emphasizing the scientific scarcity and novelty of the suggested study [14]. The goal of this bibliometric review is to evaluate international research trends on the effects of pollutants (diesel, heavy metals and microplastics) on the penguins native to Antarctica and closely related topics. In this study, the bibliographic database was built from sources extracted from Scopus. Bibliometric data in a 20-year time frame (2000–2020) were collected using the following keyword combinations from the Scopus website:

i. (diesel fuel) AND “penguins” AND (“Antarctic” OR “Antarctic”).
ii. (heavy metal) AND “penguins” AND (“Antarctic” OR “Antarctic”).
iii. microplastic AND “penguins” AND (“Antarctic” OR “Antarctic”).

The search was limited to articles pertaining to only these keywords in order to avoid the recurrence of similar research in other forms of documents published. Based on the Scopus database query results, the total publications obtained were 3 documents on diesel, 22 articles related to heavy metals and 2 documents on microplastic research. To provide a comprehensive and in-depth overview, bibliometric software (VOSviewer, version 1.6.15), interactive mapping software (Tableau Public) and an online chart illustrator (Datawrapper) as well as Microsoft Excel for data organization were used. These tools provide a visual mapping of the current trends and research development, focusing on the analysis of the link strength of networks based on the topographical distribution, chronological research trends, subject areas, source journals and network of keyword occurrences [15,16].

**Figure 1:**
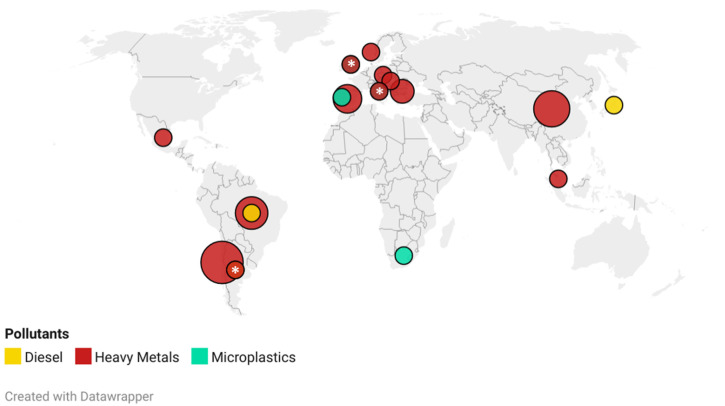
Distribution of article publications categorized by diesel, heavy metals and microplastics pollution affecting penguins in Antarctica. The interactive map can be accessed by following the link: https://datawrapper.dwcdn.net/4FOSI/2/. (accessed on 2 May 2021). Note: overlapped countries indicated by asterisk (*).

Articles published on the effects of diesel, heavy metals and microplastics pollution towards penguins in Antarctica are illustrated in Figure 1. The number of articles contributed by each country is represented based on the authors’ affiliation. A substantial percentage of articles published on heavy metals affecting penguins were from Chile (32%), followed by China (23%), Brazil (18%), Spain (14%) and Bulgaria (9%), while other countries (Argentina, Czech Republic, Hungary, Italy, Malaysia, Mexico, Norway and United Kingdom) had contributed equally (5%). In 1959, countries including Argentina, China, Chile, Norway and the United Kingdom signed the Antarctic Treaty as a framework for environmental protection that committed them to protect and conserve Antarctica [17]. Thus, an appreciable number of studies on the detrimental effects of heavy metal pollution on penguins in Antarctica were performed in the past two decades. In comparison, studies on diesel (3 countries) and microplastic (4 countries) pollution affecting Antarctic penguins were conducted by fewer countries. The dominance of certain countries on these research topics could be due to the many Antarctic research stations built by them, with Chile having 12 active stations, China with 4 research stations and Brazil with 1 station. Recent advancements in polar research have linked international collaborators (researchers, academicians, postgraduate students) from different parts of the globe. The substantial amount of financial support, ease of monitoring and data collection contribute further to the increase in research activities in Antarctica [18,19]. Great efforts by all nations are essential in tackling and mitigating pollution in the Antarctic environment, as stipulated by the Protocol on Environmental Protection to the Antarctic Treaty (Annex II: Flora and Fauna).

In this review article, the background of penguins in Antarctica, the anthropogenic pollution and cases as well as diesel, heavy metals and microplastics toxicities and their impacts on penguins, together with the bibliometric analysis, are discussed to highlight the research gaps that need to be addressed in the future.

## 2. Penguins in Antarctica

**Figure 2:**
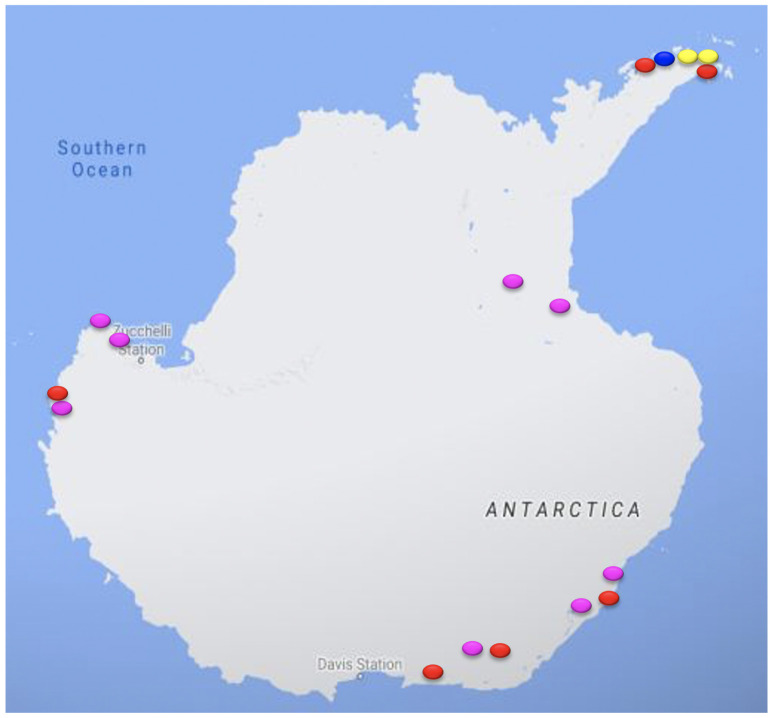
Distribution of breeding sites for four species of penguins in Antarctica [23]. The species are indicated by different colored circles; Adelie penguin (red), chinstrap penguin (blue), gentoo penguin (yellow) and emperor penguin (purple).

There are four main species of penguins that breed in Antarctica, which are the Adelie (*Pygoscelis adeliae*), chinstrap (*Pygoscelis antarcticus*), gentoo (*Pygoscelis papua*) and emperor (*Aptenodytes forsteri*) penguins. According to Sander et al. [20], 95% of the breeding community’s biomass in Admiralty Bay, Antarctica, comes from the Adelie, chinstrap and gentoo penguins. The colonies of emperor penguins are small compared to other species [21]. Gentoo and chinstrap penguins were found to be highly aggregated, with no migration data [21]. Roosens et al. [22] stated that Adelie penguins are a non-migratory species, as they spend their whole life in the Antarctic continent. The most distributed areas for breeding sites of the four main penguin species in Antarctica are in the Antarctic Peninsula and associated islands (Figure 2).

All of the Antarctic penguins have a similar body shape, but males are heavier and larger than females [24]. Penguins are monogamous animals that mate with a single partner annually and lay small eggs depending on their body weight [24]. After the breeding season, adults generally molt and are no longer waterproof and fully insulated. The penguins vary considerably in the biological characteristics of diet, height, body mass, colonies, breeding range and percentage of mate fidelity (Table 1).

Penguins play the most important role in Antarctica’s bird biomass. Owing to their importance, they can be used as biological indicators in the Antarctic polar region [28,29,30,31,32]. Biological indicators are living organisms that are used to measure the health of the environment. This is important as each individual in the biological system can give indications of the environmental health of its surroundings [33]. The penguins are suitable as biological indicator species because they are abundant, wide-ranging, easily sampled, philopatric and long-living, with relatively small foraging areas [34,35]. Their wide geographic distribution and well-defined foraging habitats also make them excellent indicators of regional pollution [36]. Penguins have the advantage of being relatively sedentary, which can reflect the local conditions as compared to other more transient bioindicator species [35].

Penguins can eliminate heavy metals through their excrement and feathers [37]. To monitor heavy metals in penguins, excreta and feathers have been frequently used due to their less invasive collection method, ease of sampling and the need for only low-cost equipment [24,25,34,37]. In fact, penguins molt annually, thus making their plumage a perfect indicator of bioaccumulation [38,39]. The level of heavy metals in the penguin’s plumage can reflect the level in the blood due to the feathers being connected to the blood vessels and trace metals having a high affinity for the sulfhydryl groups of keratins in the penguin feathers [38,40].

## 3. Pollution Incidents and Consequent Impacts on Penguins

**Figure 3:**
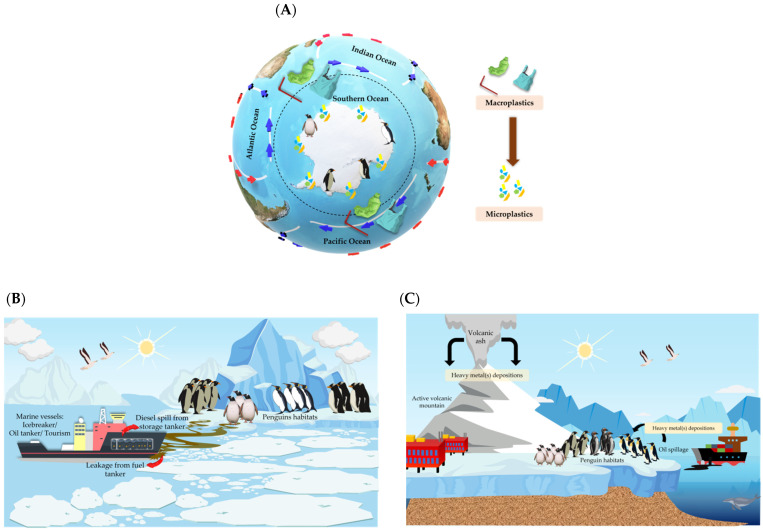
Sources of (**A**) microplastics, (**B**) diesel and (**C**) heavy metals pollution in Antarctica and the effects towards penguins.

Pollution in the cold Antarctic temperatures has detrimental effects on penguins even upon low exposure to pollutants (Table 2). Survivors of the initial pollution suffer from short- and long-term chronic effects [41,42]. Even though oil spill cases are relatively low in the Antarctic, incidents that happened near the penguins’ habitats and breeding grounds can exert a major impact on the penguins, especially during the breeding season, when the seabirds are under significant nutritional stress. This shows that penguins are more susceptible to pollutants as compared to other birds in Antarctica (Figure 3).

The harmful effects caused by pollutants towards the penguins vary according to the route of exposure for the different types of pollution. For diesel and heavy metal pollutants, bioaccumulation and direct exposure such as ingestion or direct contact can pose harm to the penguins [4,49]. Even though microplastics leach toxic chemical compounds into the environment, it is usually more harmful when the penguins physically ingest microplastics, which can cause suffocation, choking and stomach congestion as a result of plastics entanglement [50].

While oil spills and heavy metals originate from local contamination, there have been many studies that have reported large amounts of plastic pollution being carried from other regions through transport vectors, which are the global ocean current and wind [48,51]. Although it is clear that the number of visitors entering the region has significantly increased the amount of plastic pollution, there is no doubt that plastic pollution can be transported across thousands of miles from different regions to Antarctica [51].

## 4. Diesel Pollution

Demand for diesel in Antarctica is high as it provides fuel for vehicles and electricity generation in research stations and other activities concentrated at the land areas [52]. The diesel required is supplied from neighboring countries such as Australia (Hobart region) and Chile (Punta Arenas region). The transportation and storage of diesel fuel in bulk quantities can significantly increase the risk of pollution from events such as accidental spills and leakages [53]. In 2010, three 200-liter drums of diesel fuel were released from a helicopter carrying supplies from Davis Station in an effort to maintain flight stability. Consequently, the oil drums ruptured and contaminated the sandy soil near Lake Dingle. A total of 168 tonnes of contaminated soil required clean-up in an effort that took nearly 4 years to complete [54]. Such accidental events would be more devastating if they were to occur over the Antarctic waters, as the diesel would not be contained and clean-up would be more challenging.

### 4.1. Antarctic Diesel Fuel—Composition

There is a wide range of diesel fuel products developed for a variety of applications. Generally, diesel is composed of a complex hydrocarbon mixture obtained from fractional distillation and catalytic cracking of petroleum crude oil [55]. The main complex mixture of aliphatic and aromatic hydrocarbons in diesel consists of 25% to 50% of alkanes, 20% to 40% of cycloalkanes and 10% to 57% of aromatic contents [4,56]. Depending on the specifications, diesel products are differentiated by several key characteristics, such as density, viscosity, flash point, pour point, cetane number/calculated cetane index, water content and sulfur content. In 2020, greater emphasis was given to the ecological impact of diesel fuel emission, with the implementation of a new regulation for lower global sulfur cap, and for a more stringent Sox emission control. Prior to this, a number of sea areas were designated as Emission Control Areas (ECAs), which prohibit ships from operating on diesel fuel with more than 0.1% sulfur content.

For applications in Antarctica, diesel fuels are formulated with specifications for extreme cold conditions. Large vessels in Antarctic waters operate using Intermediate Grade Fuel Oil (IFO 180) and Marine Gas Oil (MGO) [4]. The type of diesel mostly used in the Antarctic and sub-Antarctic region is light grade fuel known as Special Antarctic blend (SAB) diesel, due to its low density and viscosity, which are needed to prevent its solidification at low temperatures [57,58]. A comparison of the different types of diesel fuel used in Antarctica and the sub-Antarctic region is provided in Table 3.

### 4.2. Diesel Toxicity Effects on Penguins

The SAB, MGO and IFO 180 diesel fuel contain varying hydrocarbon compositions that result in varying toxicity levels towards the ecosystem. When in contact with water, different hydrocarbon compositions of diesel fuel can result in different degrees of water-accommodated fraction (WAF) ability [5]. The impact of oil spillage depends on the bioavailability of WAF towards marine organisms. Lim et al. [62] stated that MGO and IFO 180 are generally more toxic towards marine organisms than SAB upon longer exposure, while the SAB diesel has the highest magnitude of acute toxicity upon short-term exposure.

Penguins are flightless seabirds that swim but often need to resurface to breathe, usually in a group and, thus, are at risk of harm due to diesel pollution. However, the severity of the effects depends on the species, amount of exposure and type of oil. In addition, penguins can accumulate polycyclic aromatic hydrocarbons (PAHs) through the food chain and during preening [63,64]. Penguins that are contaminated with a low concentration of petroleum can exhibit hazardous effects, such as lowered circulating hormones, increased corticosterone in females and suppressed breeding [11,65]. Even a small quantity of oil on the plumage of the penguins can result in waterlogging, thus reducing their buoyancy, thermal insulation and waterproofing, leading to hypothermia and dehydration [66,67]. Histopathological changes to the liver, kidneys and intestine, osmoregulatory impairment and hemolytic anemia, loss of abdominal and subcutaneous fat, as well as higher than normal nematode infestations and stomach ulcers can be developed after ingestion of oil, even in low quantities [66].

Meanwhile, Troisi et al. [68] detected PAH metabolites in liver samples from oiled seabirds, with concentrations in the range of 0.04–0.97 µg/g, beyond the level observed in environmentally exposed seabirds via the food chain. According to Taniguchi et al. [69], who studied PAHs in the fat tissue of penguins in Antarctica, the PAH distribution encountered in the fat samples is similar to the petroleum derivatives in Diesel Fuel, Arctic (DFA) grade. Moreover, the PAHs present in diesel can affect the cell-mediated response of the birds to immunogens, thus resulting in immunotoxic conditions [68]. The accumulation of PAHs in the bile and urine for subsequent excretion by seabirds such as penguins is due to their mixed-function monooxygenase enzyme system, which converts PAHs into water-soluble metabolites [70]. The presence of PAH metabolites in vertebrates due to exposure to oil spills has been reported in many studies [71,72]. Penguins exposed to hydrocarbon pollution may develop a reduction in fertility, low egg production, low hatching rates and embryotoxicity, which affect their reproductive performance [73].

The survival rates of oiled seabirds after being affected by oil spills depends on the degree of oiling, condition of plumage and duration of oil exposure [66]. According to Troisi et al. [74], when the seabirds are covered with oil, they cannot forage for food as they are unable to dive. Oiled penguins show a significant decline in mass as their metabolic rate is greater than non-oiled birds, which can lead to accelerated energy consumption that would eventually lead to death [75]. The metabolic rate is greater due to the increased heat loss caused by reduced thermal insulation as a result of oil affecting the penguins’ plumage [76]. Penguins that are exposed to hydrocarbons can also die due to poisoning or narcotic-like intoxication [77]. According to Barreto et al. [70], among 75% of oiled penguin cases, lung congestion was the highest histopathological finding, following hepatic congestion, found in dead penguins.

## 5. Heavy Metal Pollution

Heavy metals are a natural component in the environment but are also released through human activities. In Antarctica, high levels of heavy metals have been detected in biotic samples from terrestrial and aquatic organisms, as well as in abiotic samples such as soil, snow and atmosphere [78]. The Antarctic Peninsula has a high amount of volcanic activity that can naturally release heavy metals, while algal blooms can also explain the presence of heavy metals [40]. Anthropogenic sources such as sewage, mining, oil spills, abandoned dump sites and pesticides can also pollute the environment with heavy metals. Heavy metal pollutants have been reported in the vicinities of Russian Antarctic stations [79], freshwater lakes of Thala Hills [80], surface soils around Syowa station [81] and Casey Station [82].

Previous research suggested that heavy metals can be transported from other continents in the southern hemisphere to Antarctica via long-range atmospheric transport [83]. In Deception Island, Hg levels detected in water and sediments were up to 10,000 times higher than the other site, possibly attributed to volcanic activity [84]. Similarly, levels of Cu, Pb and Zn measured from Thalla Valley tip, an abandoned waste disposal site near Casey Station, also showed elevated levels from 100 to 1000 times greater than those in non-impacted locations [85].

In addition, ornithogenic soils that result from the deposition of guano by marine birds can be a source of heavy metal pollution [86]. Heavy metals are transported to the terrestrial from the marine ecosystem through the guano of penguins. According to Chu et al. [47], the amounts of Cu, Zn, Pb, Cd and Hg heavy metals on Ardley Island are higher after being contaminated with penguin guano. According to Evans et al. [87], high contents of Cd, Cu and As have also been reported in orinthogenic soils from penguin rookeries in the Antarctic Peninsula and South Shetland Islands. Carcasses of animals such as seals are the other contributor to the increasing level of heavy metals in Antarctic soil. Carcasses has been shown to contribute a significant source of Hg [88]. Based on information obtained from a previous study, Cu, Pb, Zn, Cd, Hg and As are the most noted sources of heavy metals contributing to local contamination in Antarctica [89].

The presence of high concentrations of heavy metals in Antarctica is an emerging issue (Table 4). Although some studies have found that the amounts of heavy metal toxicity in the feathers of penguins are still below the acceptable limits, continuous exposure to heavy metals even at low amounts could affect the endemic species in the long term [90,91].

There are many adverse effects of heavy metals on penguins. The toxicity of heavy metals can lead to severe damage of the penguin’s kidneys, liver and central nervous system [92]. The biotoxic mechanism of heavy metals such as Cd is through their ability to strongly bind to metallothioneins, which are proteins in the membranes of cell organelles and in the kidneys of penguins [92]. The accumulation of Hg can also affect the behavior and reproduction systems of penguins, causing lower fecundity and hatching rate, decreased egg size and increased mortality [94]. In addition, the uptake of Hg in penguins can reduce their food intake and lead to weight loss, thus resulting in body weakness [24].

The effects of the accumulation of heavy metals in penguins can spread to the environment as well. Chu et al. [47] mentioned that the higher heavy metal concentration in the sediment of Ardley Island, Antarctica is due to penguin guano. As penguins can transport contaminants from ocean to land through geo–biological–chemical circulation, they have a tendency to spread pollution to the environment [47,95].

## 6. Microplastic Pollution

Throughout the globe, plastic pollution is ubiquitous. Evidence of plastic pollution also has been found across the most remote islands and beaches in Antarctica, which have never been visited before [96,97,98]. The oceanic islands in the Southern Ocean can be considered amongst the most remote shores as they are uninhabited and geographically isolated. The island shores survey in Scotia Arc, Southern Ocean revealed the significant presence of marine debris on the beaches [96,97]. Previous research has suggested that the plastic debris present in the remote areas is transported from distant sources by ocean currents and local shipping [97]. The types of marine plastic debris found on beaches in remote polar areas are listed in Table 5.

As a pollutant, microplastics have gained much notoriety in recent years as they are pervasive and persistent. Microplastics are defined as plastic particles that are smaller than 5 mm, either originally manufactured as such (primary source) or as the fragmentation products of larger plastics (secondary source) [99]. Microplastics from the breakdown of plastic debris are common in oceans throughout the world and persist both in surface and deep ocean waters [100]. These plastics undergo fragmentation until they reach nanometer sizes or mineralize into biomass and carbon dioxide [101]. Microfibers are the main microplastic components that are commonly found in oceanic surface waters [102,103].

In Antarctica, the sources of plastic pollution are fishing activities, tourism and research stations [103]. Recently, microplastic polymers have been detected in an ice core sampled from coastal land-fast sea ice in East Antarctica [104]. Moreover, microplastic pollution has been reported in intertidal sediments from the sub-Antarctic island of South Georgia [100], coastal environment of Potter Cove [105], intertidal region of the South Shetland Islands [106], terrestrial environment of Signy Island [107], sea ice of Bellingshausen Sea [108] and around Union Glacier and Antarctic Plateau [109]. Concentrations of microplastic pollution were reported to be the highest in these areas, mainly due to the high density of maritime traffic and the high number of research stations located within the vicinity of the Antarctic Peninsula/Scotia Sea region [100]. In a study conducted by Fragao et al. [110], the authors reported microplastic as the main anthropogenic particle found in Adelie, Chistrap and Gentoo penguins.

Microplastics can be ingested via direct ingestion or indirect ingestion. For direct plastic debris ingestion, penguins are not strongly impacted as they target live prey and do not pay attention to the floating plastic [36]. When the plastic is ingested, it can cause gut inflammation, and microplastics are able to penetrate the digestive tract barrier to reach the blood and organs, thus affecting their function [103]. However, plastic from fishing gear entanglement has been reported in 7 of 18 penguin species [103]. Both ingestion or/and entanglement of plastic debris will lead to higher mortality risk [111]. Chemicals from the plastics may leach faster into penguins’ stomachs than into seawater [112]. Leaching also enhances the adhesion of hydrophobic waterborne pollutants onto the hydrophobic surface of the plastic. As a result, this can introduce harmful substances into food webs as microplastics persist for a long time when ingested. Other than chemicals, microfibers contain plastic additives such as dyes or flame retardants that increase toxic bioavailability to organisms [103].

Microplastics are introduced into the marine food chain via low trophic level organisms [103]. An example of such an organism is the Antarctic krill, *Euphausia superba*, which was shown to ingest microplastics available in the sea. A recent laboratory study has proven that Antarctic krill are able to convert microplastic particles into nanoplastics through digestive fragmentation [101,113]. This krill is a common diet of *P. adeliae*, *P. antarcticus* and *P. papua* (Table 1) [99]. Other studies reported the presence of microplastics in mesopelagic fish from the North Pacific [114,115] and North Atlantic Oceans [116,117]. At the base of the food chain, the impact of microplastics is extensive, disrupting digestive tracts, creating false food fullness or transferring toxic compounds that interrupt food availability for penguins [103].

Microplastic fibers and fragments were found in the excrement of gentoo penguins due to indirect microplastic contamination via the food web [99,118]. Bessa et al. [99] found the presence of microplastics in the gastrointestinal tracts of penguins feeding in Antarctic waters. A study by Lourenco et al. [119] showed that the guano of invertebrates and shorebirds in the Eastern Atlantic Ocean contains similar microfiber compositions, which were mainly ingested from their prey. Microplastic pollution can cause harm to wildlife as well as facilitate the transport of non-indigenous species [120].

## 7. Bibliometric Analysis

**Figure 4:**
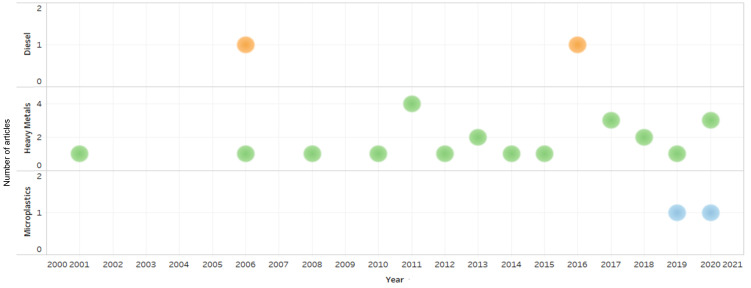
Chronological trend of published research articles on diesel, heavy metals and microplastics pollution among penguins in Antarctica from 2000 to 2020.

The annual number and growth of published articles from 2000 to 2020 on the toxicity of various pollutants towards penguins are shown in Figure 4. Significant growth was observed for the topic of heavy metal pollution from 2001 till 2020, while diesel pollution (2006 and 2016) and microplastics (2019–2020) pollution were the focus of far fewer articles. This indicates that the topic of heavy metal toxicity towards penguins was frequently investigated and sustained great interest among the scientific community. This trend is expected to continue, followed by microplastic and diesel pollution and their toxicities in penguins.

**Figure 5:**
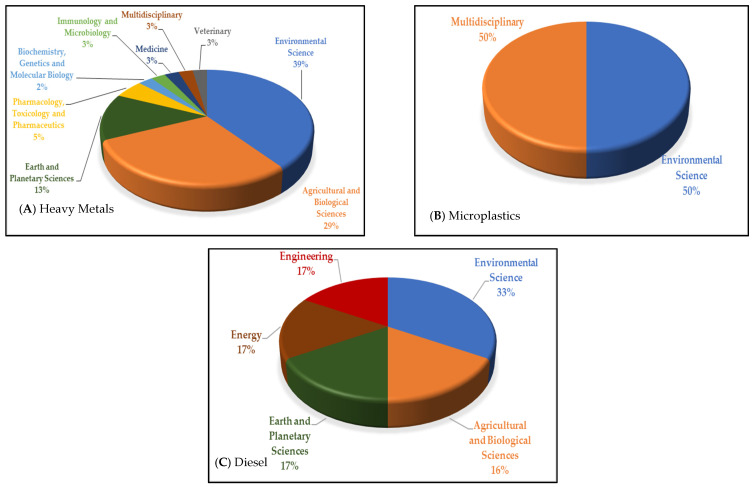
Top subject areas on the research of heavy metals (**A**), microplastics (**B**) and diesel (**C**) pollution and their effects on penguins indigenous to Antarctica.

A broad range of subject areas contributed to the published articles on the effects of pollution toward penguins in Antarctica (Figure 5). According to the data analysis, the top three subject areas on heavy metal pollution among penguins (Figure 5A) are environmental science (39%), agricultural and biological sciences (29%) and Earth and planetary sciences (13%). A similar pattern was seen for the topic of diesel pollution among penguins (Figure 5B), with environmental science (33%), agricultural and biological sciences (17%) and Earth and planetary sciences (16%). Meanwhile, research papers on microplastic pollution towards penguins are evenly divided (50%) between environmental science and multidisciplinary areas (Figure 5C). Environmental science is an interdisciplinary study area that is associated with biological and physical disciplines to identify environment-related problems and solutions, such as examining the chemical effects of toxic pollutants on the biota or the bioremediation methods employed to remediate oil spills [121]. The agricultural and biological sciences subject area consisted of articles that investigated the accumulation of pollutants (heavy metals, diesel and microplastics) in penguins that were detected in the body or soil through the food chain, with plants as the primary producers [122]. These subject areas are important supporting areas of research on the toxicity of heavy metal pollution among penguins. The Earth and planetary sciences were the third most popular subject area on the topic of heavy metals and diesel. Moreover, multidisciplinary subject areas included assorted scientific disciplines or specializations. The toxicity effects of such pollutants are severe among Antarctic fauna, specifically penguins; hence, they are being actively studied. Our analysis shows that the research on the effects of heavy metals and diesel in penguins is more widely explored compared to microplastics under various disciplines. The research authors’ expertise also has an impact on the categorization of papers into distinct subject groups, which is important for this evaluation and future research.

**Figure 6:**
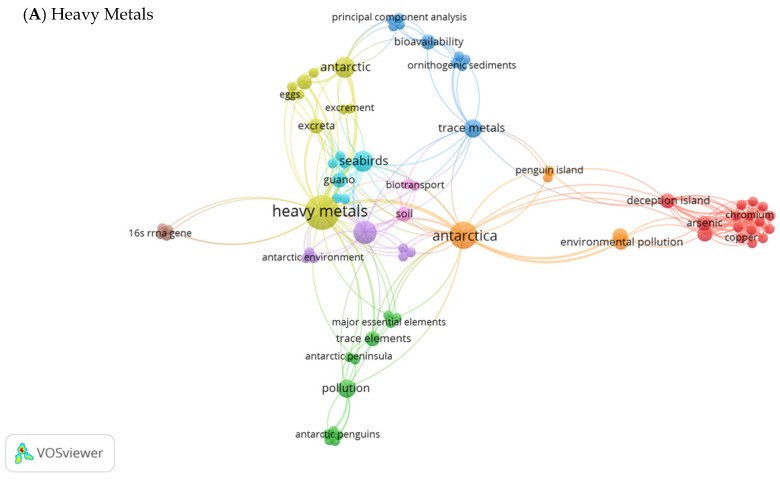

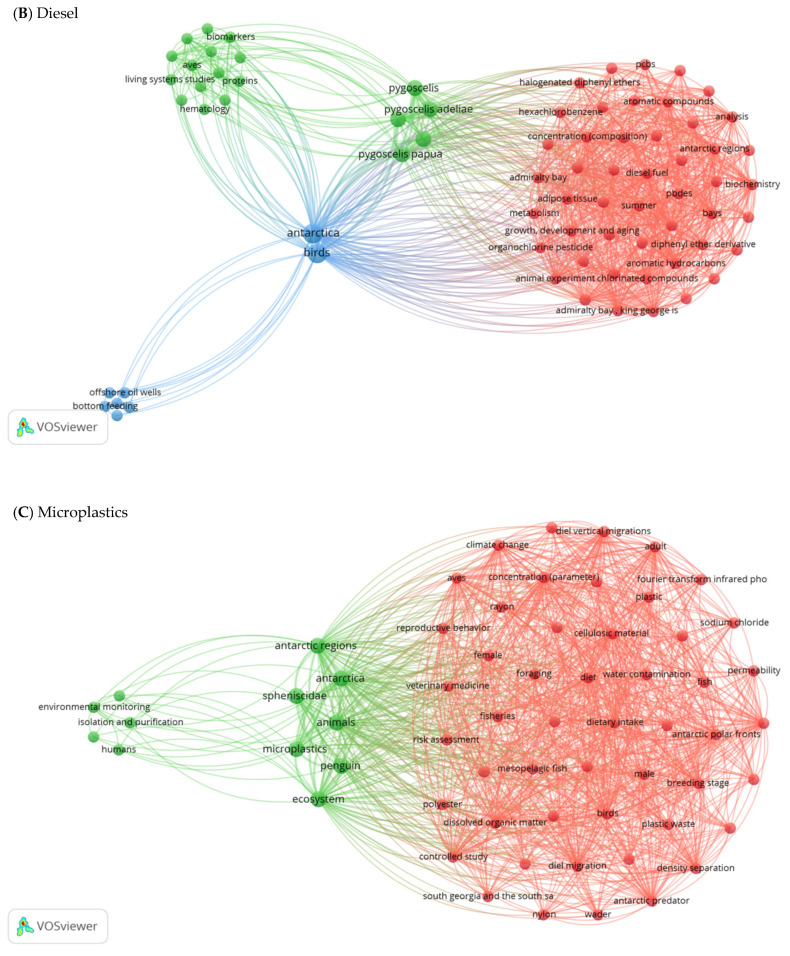
Visualization map of keyword analysis network on the impacts of pollutants towards penguins in Antarctica. (**A**) Heavy metals—clusters were distributed as follows: Cluster 1 (red) 16 items, Cluster 2 (green) 12 items, Cluster 3 (blue) 10 items, Cluster 4 (yellow) 10 items, Cluster 5 (purple) 7 items, Cluster 6 (light blue) 7 items, Cluster 7 (orange) 5 items, Cluster 8 (brown) 4 items and Cluster 9 (pink) 3 items. (**B**) Diesel—clusters represented as Cluster 1 (red) 48 items, Cluster 2 (blue) 19 items and Cluster 3 (green) 8 items. (**C**) Microplastics—clusters formed are displayed as Cluster 1 (red) 50 items and Cluster 2 (green) 12 items.

Keyword network analysis is a vital process to identify the current hot topics in research [123]. Network maps illustrate the strength of co-occurrence that links a pair of keywords in varying line thicknesses, while the size of the keyword’s label or node represents the occurrence of those keywords in publications. Figure 6A illustrates the occurrence of keywords in all the articles published on heavy metal toxicity towards penguins in Antarctica. From this analysis, the overall total strength (TLS) was 354, with the total number of links of 332 among the 9 clusters formed. The clusters were dominated by the central keywords, “heavy metals”, “antarctica”, “penguins”, “trace metals”, “pollution”, “seabirds” and “soil”. Different levels of heavy metals (arsenic, lead, chromium, nickel, vanadium, copper, zinc and strontium) were known to be accumulated in many penguin species, including the gentoo, chinstrap, guano and Adélie, scattered along Deception Island, King George Island, Livingston Island and Penguin Island (South Shetland Islands) in Antarctica. Hence, strong interactions were identified within the keywords that occurred in the topics. According to the network analysis, the biotransportation of heavy metals or trace metals was identified from penguins’ droppings or excrement in Antarctica.

The keyword analysis formed three clusters based on the impacts of diesel on penguins in Antarctica, with 1701 links and a TLS of 1723 (Figure 6B). The keywords “birds” and “Antarctica” were closely related and centralized in the cluster network. Moreover, the hydrocarbons displayed in the visualization map included PAHs, polychlorinated biphenyls (PCBs), persistent organic pollutants (POPs) and polybrominated diphenyl ethers (PBDEs). In addition, the link between hydrocarbons such as PAHs to birds shows that these two topics are related, as some publications explained the toxicity of hydrocarbons to penguins since they are common bird species in the Antarctic. Besides penguins, sheathbills, south polar skua and gulls are some other bird types in Antarctica that are also at risk. Only two clusters (Figure 6C) were constructed from 62 identified keywords, with a TLS of 1662 and total links of 1641. The keywords “Antarctic regions” or “Antarctica”, “spheniscidae” or “penguin”, “ecosystem”, “microplastics” and “animals” were presented as the focus of Cluster 2, with equal strength of connected links. Different sources of microplastics such as rayon, polyester, plastic, fibers or microfibers, cotton (*Gossypium hirsutum*) and nylon were reportedly found among the penguins that breed along the coastline and feed on fish contaminated by microplastics.

## 8. Conclusions

Adelie, chinstrap, gentoo and emperor penguins are the main species of penguins that breed in Antarctica. Due to these three species being widely distributed in Antarctica, they are prone to exposure to environmental pollutants such as diesel, heavy metals and microplastics. The bibliometric output showed that 27 articles related to the toxicity of pollutants (diesel, heavy metals and microplastics) were published and indexed in the Scopus database from the year 2000 to 2020. The topic of heavy metal toxicity was the most emerging research area regarding its risks among penguins. Several limitations contributed to the low number of articles retrieved since our search was limited to the Scopus database for the analysis. The use of other journal databases such as the Web of Science and Google Scholar might return a higher number of articles. Nevertheless, the low number of published articles on the topic of diesel and microplastics in comparison to heavy metal pollution indicates that they are relatively unexplored research topics for emerging contaminants in Antarctica that arise from growing human activities. With the adverse toxicity effects of pollution towards penguins, these pollutants should be monitored and their use restricted. Therefore, studies about the toxicity of pollutants toward penguins are highly encouraged in order to preserve the delicate ecosystem balance in Antarctica.

## Figures and Tables

**Table 1:** Some biological characteristics of Antarctic penguins.

Type of Penguins	Diet	Height (cm)	Mean Body Mass (kg)		Mate Fidelity (%)	Breeding Range (°S)	Colonies
			Male	Female			
*Pygoscelis adeliae*	Feeds mainly on Antarctic krill (*Euphausia superba*) [25]	70 [24]	5.6 [24]	4.9 [24]	80 [24]	54–77	Significantly clustered [21]
*Pygoscelis antarcticus*	Feeds almost exclusively on Antarctic krill (*Euphausia superba*), lower amounts of Antarctic silverfish (*Pleurogramma antarcticum*) and very low amounts of amphipods [24]	71–76 [24]	5.0 [24]	4.7 [24]	83 [24]	54–64	Highly aggregated [21]
*Pygoscelis papua*	Feeds on Antarctic krill (*Euphausia superba*), fish and small squid [26]	75–90 [24]	5.6 [24]	5.2 [24]	85 [24]	46–65	Relatively evenly spaced [21]
*Aptenodytes forsteri*	Feeds entirely on fish (*Pleuragramma antarcticum*), cephalopods and crustaceans [27]	100–130 [24]	37.3 [24]	28.8 [24]	5 [24]	66–78	Significantly clustered and less aggregated [21]

**Table 2:** Pollution incidents and their effects on penguins in Antarctica.

Pollution	Incident	Effect(s) on Penguins	Reference (s)
Oil	*Bahia Paraiso*, near southern Anvers Island in January 1989	Up to 300 dead oiled seabirds, mostly Adelie penguins (*P. adeline*), were found.	[43,44,45]
	*Cape Hallett*, Ross Sea in January 2001	50–100 of oiled Adelie penguins.	[45]
	*MS Explorer* in November 2007	Around 2500 penguins affected as the spill site included the breeding grounds for Adelie penguins and the largest mating colony for Papua penguins.	[45,46]
	*Oliva* in Nightingale Island, Tristan da Cunha Group in March 2011	Several landing sites of penguins were covered in oil; 3650 penguins in holding pens, 373 died and 3800 penguins were captured for rehabilitation.	[45]
Heavy Metals	The concentrations of Cu, Zn, Pb, Cd, Hg and P are significantly higher in Ardley Island, Antarctica	Study of various trace elements in feathers, eggs and excreta of gentoo penguins indicated that the element concentrations are highest in the excreta.	[47]
Plastics (Microplastics)	Plastic bottles and other labeled marine debris manufactured in South America have already been identified both within the Scotia arc and outside the Polar Front at Marion Island, Antarctica	Plastics were incidentally identified in the stomach contents of penguins inside the Polar Front and in the Weddell, Ross and Scotia Seas.	[48]

**Table 3:** Comparison of different types of diesel fuel used in Antarctica and sub-Antarctic region.

	Special Antarctic Blended Diesel (SAB)	Marine Grade Oil (MGO) [59]	Intermediate Grade Fuel Oil (IFO 180) [60]
Oil type	Distillate	Distillate	Blend of distillate and residual oil
Production process	Straight run refinery	Straight run refinery	Straight run refinery and catalytic cracking process
Composition	n-alkanes (C_9_–C_14_), branched alkanes, cyclic alkanes and aromatic hydrocarbons [61]	n-alkanes (C_9_–C_25_), cyclic alkanes, aromatic hydrocarbons	n-alkanes (C_6_–C_40_), branched alkanes, cyclic alkanes, aromatic hydrocarbons, polycyclic aromatic hydrocarbons
Color	Colorless	Colorless to brown	Black
Kinematic viscosity (mm^2^/s)	<7.0 at 40 °C	1.5–6.0 at 40 °C	180 at 50 °C
Flash point (°C)	>61.5	>61.5	>61.5
Pour point (°C)	<−35	<−6	<30
Density (g/cm^3^) at 15 °C	0.81	0.84	0.99
Solubility in water	Insoluble	Insoluble	Very slightly soluble

**Table 4:** Higher concentrations of heavy metals found in Antarctica.

Location	Heavy Metals	Cause	Reference (s)
O’Higgins	Hg Pb	Higher concentration of human activities (shipping, boating and loading and unloading of fuel and goods)	[92]
Vindela	As Cd	Strongly related to local volcanism in the area under study	[92]
Antarctic Peninsula	As	Concentrated volcanic activity	[93]
King George Island	Cu Zn	Intercontinental atmospheric transport and fuel utilized	[34]
Ardley Island	Cd Cu Zn	Contaminants are transported to the lacustrine sediments in the form of penguin guano after a series of biomagnification in the food chain	[47]
O’Higgins Base, Stranger Point, Neko Harbor and Doumer Island	Cd Co Cr Mo Ni V	Large-scale mining activities	[40]

**Table 5:** Beached marine debris found in remote areas in Antarctica.

Location		Type of Debris	Countries of Manufacture	Reference (s)
		Plastic bottle	Argentina, Chile	
South Georgia	Bird Island	Fishing net	-	[98]
		Plastic rope	-	
		Packaging bags	-	[97]
		Polystyrene	-	[97]
	Husvik	Fishing net	-	[96]
		Plastic rope		
		Plastic bottle	Argentina, Chile, Japan	
South Sandwich	Candlemas Island		-	
		Fishing net	Russia	
		Other plastic		[96]
			Argentina	
	Saunders Island	Plastic bottle	-	
		Fishing net	-	
		Plastic rope		
		Plastic bottle	South America	[98]
		Fishing net	-	
South Orkney	Signy Island	Plastic rope	-	
		Packaging bags	-	[97]
		Polystyrene	-	[97]

## Data Availability

Not applicable.
